# Ceramide and palmitic acid inhibit macrophage-mediated epithelial–mesenchymal transition in colorectal cancer

**DOI:** 10.1007/s11010-020-03719-5

**Published:** 2020-03-28

**Authors:** Raimundo Fernandes de Araujo Junior, Christina Eich, Carla Jorquera, Timo Schomann, Fabio Baldazzi, Alan B. Chan, Luis J. Cruz

**Affiliations:** 1 Department of Morphology, Federal University of Rio Grande do Norte, Natal, RN 59072-970 Brazil; 2 Post-Graduation Programme in Structural and Functional Biology, Federal University of Rio Grande do Norte, Natal, RN 59072-970 Brazil; 3 Post-Graduation Programme in Health Science, Federal University of Rio Grande do Norte, Natal, RN 59072-970 Brazil; 4 Percuros B.V., 2333 CL Leiden, The Netherlands; 5 Translational Nanobiomaterials and Imaging, Department of Radiology, Leiden University Medical Center, 2333 ZA Leiden, The Netherlands

**Keywords:** Ceramide, Palmitic acid, Macrophage 1, Macrophage 2, Colorectal cancer cells, Epithelial–mesenchymal transition

## Abstract

Accumulating evidence indicates that ceramide (Cer) and palmitic acid (PA) possess the ability to modulate switching of macrophage phenotypes and possess anti-tumorigenic effects; however, the underlying molecular mechanisms are largely unknown. The aim of the present study was to investigate whether Cer and PA could induce switching of macrophage polarization from the tumorigenic M2- towards the pro-inflammatory M1-phenotype, and whether this consequently altered the potential of colorectal cancer cells to undergo epithelial–mesenchymal transition (EMT), a hallmark of tumor progression. Our study showed that Cer- and PA-treated macrophages increased expression of the macrophage 1 (M1)-marker CD68 and secretion of IL-12 and attenuated expression of the macrophage 2 (M2)-marker CD163 and IL-10 secretion. Moreover, Cer and PA abolished M2 macrophage-induced EMT and migration of colorectal cancer cells. At the molecular level, this coincided with inhibition of SNAI1 and vimentin expression and upregulation of E-cadherin. Furthermore, Cer and PA attenuated expression levels of IL-10 in colorectal cancer cells co-cultured with M2 macrophages and downregulated STAT3 and NF-κB expression. For the first time, our findings suggest the presence of an IL-10-STAT3-NF-κB signaling axis in colorectal cancer cells co-cultured with M2 macrophages, mimicking the tumor microenvironment. Importantly, PA and Cer were powerful inhibitors of this signaling axis and, consequently, EMT of colorectal cancer cells. These results contribute to our understanding of the immunological mechanisms that underlie the anti-tumorigenic effects of lipids for future combination with drugs in the therapy of colorectal carcinoma.

## Introduction

Colorectal cancer is among the most prevalent cancers in both genders. Each year, approximately, 1.36 Mio patients are diagnosed with colorectal cancer worldwide and ∼700.000 patients die because of this malignancy [1]. Treatment resistance and regrowth of tumors after chemo- or radiotherapy pose a major threat to the survival of cancer patients. New therapeutic strategies are, therefore, urgently needed to delay tumor growth and to prevent metastasis.

Malignant tumors are a complex assembly of genetically heterogeneous cancer cells and different cell types that constitute the local tumor microenvironment. These cells include endothelial cells, cancer-associated fibroblasts, and different populations of immune cells. Tumor-associated macrophages (TAMs) are among the most abundant immune cell types in the tumor microenvironment of solid tumors and are recruited as monocytes from the peripheral blood [2]. They express a broad spectrum of activation states between the two extreme forms of classical ‘M1′ or alternative ‘M2′ in line with the Th1/Th2 dichotomy [2–6]. Bacterial moieties, such as lipopolysaccharide (LPS), and interferon-γ (IFN-γ) polarize macrophages toward the M1 phenotype, which show pro-inflammatory, anti-microbial, and anti-tumor activities by high expression of tumor necrosis factor (TNF)-ɑ, interleukin (IL)-1, IL-6, and IL-12, reactive oxygen species, and major histocompatibility complex (MHC-II) molecules [7]. In contrast, IL-4, IL-10, and IL-13 polarize macrophages towards the M2 phenotype [8]. M2-skewed TAMs are characterized by the upregulation of various molecules, including the scavenger receptor CD163, mannose receptor (CD206), and the immunosuppressive cytokine IL-10 [9].

After entry into the tumor, monocytes preferentially differentiate into M2-skewed TAMs and promote tumor progression by suppressing anti-tumor immunity, stimulating neovascularization, tumor invasion, and subsequently metastasis [5]. The latter follows a process described during the epithelial–mesenchymal transition (EMT), during which epithelial cells lose their cell polarity and cell–cell adhesion and become mobile [10]. EMT is accompanied by reduced expression of epithelial markers, such as E-cadherin (E-cad), increased mesenchymal markers, such as vimentin [11], and activation of transcription factors, such as SNAI1 [12, 13] in cancer cells. At the molecular levels, the EMT process is governed by the master EMT transcription factor signal transducer and activator of transcription 3 (STAT3), which can be activated by IL-6 [14, 15] and/or IL-10 [16, 17]. Next to STAT3, NF-κB and downstream targets, such as SNAI1 [18], cooperate with STAT3 in promoting the progression of colon cancer metastasis by conferring apoptosis resistance and epithelial plasticity. Moreover, NF-κB and STAT3 can physically interact and cooperate in regulating the interaction of malignant cells and the tumor microenvironment [19], especially immune cells, such as TAMs.

TAMs greatly contribute to the aggressive progression of cancer by releasing cell-stimulating growth factors and cytokines, including IL-6, IL-10, and TNF-α [20]. Such factors may induce EMT and enhance metastasis [21]. Imaging data suggest that EMT is involved in the emigration of carcinoma cells from primary tumors, a process that requires the inflammatory microenvironment promoted by immune cells, including TAMs [22].

Unlike stably differentiated cells, M1 and M2 macrophages can switch phenotypes [23–25]. Thus, reprogramming M2 TAMs towards the immunogenic M1 phenotype in vivo to block tumor progression could be a promising strategy for the treatment of cancers. In this context, several studies have demonstrated the potential of lipids to modulate macrophage function and phenotype [26, 27]. Palmitic acid (PA), a saturated fatty acid, was shown to induce an inflammatory response in macrophages by amplifying LPS-mediated signaling via TLR4 [28, 29], potentially mediated by NF-κB activation [30]. Ceramide (Cer), an intracellular second messenger and membrane component [31], played a key role in mediating the synergistic effect of PA and LPS on pro-inflammatory gene expression. Increases in intracellular Cer were reported in many cell types, in response to a variety of stimuli, including inflammatory cytokines TNF-α, IL-1, and IFN-γ [32], as well as apoptosis-inducing stimuli [33–35]. More recently, C-6 Cer delivered in nanoliposomes in vivo was shown to directly reduce the number of TAMs and M2-like markers in hepatocellular cancer [36].

In this study, we investigated how PA and Cer modulate the interplay of M2 TAMs and colon cancer cells, with a particular focus on M2-promoted EMT of colon cancer cells. Our studies show that PA and Cer reduce the M2 phenotype and suppress M2-induced EMT and migration of colon cancer cells. These findings suggest that selective targeting of Cer and PA to the tumor microenvironment could reduce tumor progression and metastasis of colon cancer cells and increase the survival of cancer patients.

## Materials and methods

### Antibodies and reagents

Mouse anti-E-cadherin, mouse anti-vimentin, mouse anti-STAT3, mouse anti-NF-κB antibodies, and Hoechst were purchased from Thermo Fisher Scientific (Waltham, MA, USA). Cer (from porcine brain) was purchased from Avanti Polar Lipids (Alabama, USA), PA was purchased from Sigma-Aldrich (Darmstadt, Germany), and anti-mouse CD163- PerCP, anti-mouse KI-67-APC, anti-mouse CD68-FITC, and anti-mouse IL-10-FITC were obtained from eBioscience (San Diego, CA, USA). Recombinant mouse IL-4, IFN-δ, and anti-IL-10R inhibitor were purchased from PeproTech (Rocky Hill, NJ, USA).

For immunofluorescence labeling, purified rabbit anti-Vimentin, rabbit anti NF-κB, rabbit anti-E-cadherin, and mouse anti-STAT3, and the secondary antibodies goat anti-mouse and goat anti-rabbit Alexa® Fluor 555 (Thermo Fisher Scientific) were used. Rabbit anti-β-actin (LI-COR Biosciences, Lincoln, Nebraska, USA) were used for immunoblotting.

### Cell lines and cell culture

The murine colorectal carcinoma (CT-26) and (MC-38) cell lines and the mouse macrophage RAW 264.7 cell lines were obtained from American Type Culture Collection (Rockville, MD, USA). Cell lines were cultivated in Dulbecco’s modified Eagle’s medium (DMEM, Gibco Laboratories, Grand Island, NY, USA) supplemented with 1% antibiotics (penicillin/streptomycin) and 10% fetal bovine serum (FBS). Cells were maintained in a humidified incubator with 5% CO_2_ at a temperature of 37 °C. Twice a week, cells were passaged by removing the adherent cells with trypsin/EDTA in phosphate-buffered saline (PBS).

### Cell viability assays

MTS assay: CT-26, MC-38, and RAW 264.7 cells were cultured (3 × 10^3^ cells/well) in a 96-well plate and after 24 h, PA and Cer were added at different concentrations (i.e., 1 µM, 2.5 µM, 5 µM, 10 µM, 30 µM, 60 µM, 120 µM, 240 µM, and 480 µM) in CT-26 and RAW 264.7 cells as well as 1 µM, 2.5 µM, 5 µM, 10 µM, 30 µM and 60 µM concentrations in MC38 cells. Cer and PA were maintained in the dark at −20 °C and dissolved in ethanol. After another 24 h, the viability of CT-26, MC-38 and RAW 264.7 cells were analyzed by adding 20 μl/well of CellTiter 96 AQ_ueous_ One Solution (MTS) solution (Promega, Madison, WI, USA) and were incubated with 5% CO_2_ at a temperature of 37 °C for 3 h. Also, cell viability of MC-38 cells were studied after 48 h. Absorbance was measured at 490 nm. All experiments were performed in triplicate and the mean of the replicates was plotted. As controls, 25% DMSO (“death” control) and culture medium without drug (“live” control) were used. Hoechst labeling and flow cytometric analysis: CT-26 and RAW 264.7 cells were cultured (5 × 10^4^ cells/well) in 12-well plates under normal culture conditions for 24 h. Next, 10 µM, 30 µM, and 60 µM of PA and Cer were added and the cells were incubated for 48 h. Afterwards, the cells were detached with trypsin/EDTA in PBS and blocked with 0.5% bovine serum albumin (BSA) in PBS for 45 min, followed by labeling with Hoechst (1:100) at 4 °C for 60 min. Following a final washing step, the cells were measured with a BD LSR II (BD Biosciences, CA, Boston, USA) and analyzed with FlowJo (version 10.1; Tree Star Inc., CA, Boston, USA). Each experiment was performed in triplicate and repeated twice to assess the consistency of the results. The mean of the replicates was plotted.

### Polarization of RAW 264.7 cells towards M1 and M2 macrophages

For induction of M2-polarized TAMs, RAW 264.7 cells (5 × 105 cells/well in a 12-well plate) were cultured in complete medium with 10% FBS, supplemented with 20 ng/mL IL-4 for 24 h. After 24 h, RAW 264.7 cells were washed three times with serum-free (sf) medium and cultured for another 48hrs in sf medium supplemented with IL-4. After 48 h of serum starvation, RAW 264.7 cells were collected and used as M2-polarized TAMs. During the polarization process, RAW 264.7 supernatant was collected and used as M2-polarized TAMs-conditioned medium (CM). For induction of M1-polarized TAMs, RAW 264.7 cells (5 × 105 cells/well in a 12-well plate) were cultured in medium with 10% FBS, supplemented with LPS and IFN-γ for 24 h. After 24 h, RAW 264.7 cells were washed three times with sf medium and cultured for another 48 hrs in sf medium [37]. To assess the effect of PA and Cer on the polarization state of M2 macrophages, RAW 264.7 cells were first cultured for 24 h in the presence of IL-4 and 10% serum, as described above. After 24 h, the cells were washed three times with sf medium and resuspended in sf medium, substituted with 10 µM Cer or 10 µM PA. The cells were cultured for another 48 h in the presence of lipids. Cells morphology, as an indication of M2 polarization, was analyzed using a Telaval 31 light microscope (ZEISS, Oberkochen, Germany).

### Flow cytometry

After incubation and treatment with 10 µM Cer or PA for 48 h, RAW 264.7 cells (1.5 × 10^4^ cells/well in a 12-well plate) were collected with a cell scraper and blocked with 0.5% BSA in PBS for 45 min. To evaluate whether the cells show characteristics of M1 or M2 macrophages, RAW 264.7 cells were labeled with anti-mouse CD163-PerCP (1:1000) or anti-mouse CD68-FITC antibody (1:1000). For intracellular IL-10 labeling, cells were first fixed in 2% paraformaldehyde (PFA) for 15 min at RT, then washed and permeabilized with 0.1% TritonX-100 for 5 min, followed by incubation with blocking solution (0.5% BSA in PBS) for 45 min and anti-mouse IL-10-FITC antibody (1:1000) for 60 min at 4 °C. Following a final washing step, the cells were analyzed by flow cytometry.

### Enzyme-linked immunosorbent assay (ELISA)

After incubation and treatment with 10 µM Cer or 10 µM PA for 48 h, the supernatants were collected and levels of murine IL-10 and IL-12 were determined using an LEGEND MAX™ Mouse IL-10/IL-12 ELISA Kit (BioLegend, San Diego, CA, USA) according to the manufacturer’s instructions. Each experiment was performed in triplicate and repeated twice.

RNA isolation and quantitative real-time PCR were performed. M2 TAMs were generated from RAW 264.7 cells as described above. During the polarization process, 10 µM Cer or 10 µM PA were added for 48 h. The cells were washed with PBS and lysed in the cell culture plate using TRIzol. (Invitrogen, Grand Island, NY, USA). Total RNA content was isolated using manufactures’ protocol. Reverse transcription was performed using the M-MLV Reverse Transcriptase (Promega, Madison, WI, USA). Expression levels of *Gapdh*, *Stat3*, *Snail1*, *Nfkb1,* and *Il10* were analyzed using real-time, quantitative PCR. All real-time PCR reactions were performed using the Real-Time PCR Detection System from Biorad and all amplifications were performed using SYBR Green and PlatinumTaq (Thermofisher Scientific). Throughout the real-time PCR analysis, the identity of the products was confirmed by melting curve analysis. The ratio of the amount of target mRNA to the amount of the internal standard (Gapdh) mRNA was determined as an arbitrary unit. The following expression primers were used: forward (F) primer CTTGTCTACCTCTACCCCGACAT and reverse (R) primer GATCCATGTCAAACGTGAGCG for *Stat3*, F primer TGGCCCAGAAATCAAGGAGC and R primer CAGCAGACTCAATACACACT for *Il10*, F primer TCTGAAGATGCACATCCGAAGCCA and R primer AGGAGAATGGCTTCTCACCAGTGT for *Snail*, F primer GAAATTCCTGATCCAGACAAAAAC and R primer ATCACTTCAATGGCCTCTGTGTAG for *Nfkb1*, and F primer ACCCACTCCTCCACCTTTGA and R primer CTGTTGCTGTAGCCAAATTCGT for *Gapdh*.

### Indirect co-culture of colorectal cancer cells and M2-polarized TAMs

CT-26 cells (1 × 10^4^ cells/well) were seeded in 12-well plates (BD Bioscience) in DMEM medium supplemented with 10% FBS. M2-polarized TAMs (5 × 10^4^ cells/insert) were seeded into the upper chamber of the transwell insert with an 8 μm pore size (Corning Inc., Corning, NY, USA) in DMEM with 10% FBS. The following day, the culture inserts with M2-polarized TAMs were treated with 10 µM Cer or 10 µM PA, in the absence or presence of 1.2 µg/mL anti-IL-10R inhibitor, and cells were cultured for another 48 h in a humidified incubator with 5% CO_2_ at a temperature of 37 °C. Cells were morphologically analyzed using a Telaval 31 light microscope (ZEISS). After treatment, CT-26 cells were collected and fixed with 2% PFA, permeabilized using 0.1% Triton, followed by incubation with anti-mouse KI-67-APC (1:100) or anti-mouse IL-10-FITC (1:100) at 4 °C for 60 min, and analyzed by flow cytometry. Moreover, CT-26 cells were collected for RNA extraction with TRIzol. The cell culture supernatants were collected and analyzed by ELISA.

### Wound-healing assay

The method used for the wound-healing assay has been described previously [38]. Briefly, the CT-26 cells and MC-38 cells were seeded in 12-well plates and incubated in DMEM medium containing 10% FBS until they reached 70% percentage. Wounds were introduced to the subconfluent cell monolayer, using a plastic pipette tip and incubated with 10 μg/mL mitomycin C for 2 h to inhibit cell proliferation. Next, the CT-26 and MC-38 cells were washed with PBS twice and cultured with CM of M2-TAM and sf DMEM (ratio 1:1), together with treatment with either 10 µM CER or 10 µM PA to CT-26 cell line and 2.5 µM CER or 2.5 µM PA to MC-38 cell line. After 4 h and/or 24 h, the area of wound induction was monitored using an Olympus IX70 light microscope (Olympus, Shinjuku, Japan) equipped with a Leica DFC340 FX digital camera (Leica Camera, Wetzlar, Germany). Digital images were acquired and stored using Leica Application Suite Advanced Fluorescence (LAS AF) software (version 1.9.0). The wound area was denoted and the amount of cells, which migrated into the area of wound induction, was measured at both time points (i.e., 4 h and 24 h, using Fiji analysis software (https://fiji.sc/). Two wound areas were evaluated per experiment and the experiment was performed in duplicate.

### Transwell migration assay

Cell migration was examined with the two-chamber assay using a transwell co-culture system (Corning). CT-26 cells(1 × 10^4^ cells)were seeded into the upper chamber of a transwell culture plate (insert) and RAW 264.7 cells (5 × 10^4^ cells) were seeded on bottom of twenty-four-well plate and incubated with DMEM medium containing 10% FBS for 24 h. Afterwards, CT-26 cells was incubated with sf DMEM and treated with 10 µM CER or 10 µM PA for 48 h. For nuclear staining, the cell suspension in the upper chamber was aspirated and the upper surface of the filter was carefully cleaned with cotton plugs, to remove all cells that did not migrate through the membrane. The nucleus of the cells that migrated through the membrane was stained with DAPI (Thermo Fisher Scientific). Three representative images of two technical replicates were taken with a Leica DM5500 B fluorescence microscope (DAPI filter), equipped with a Leica DFC365 FX digital camera. Digital images were acquired and stored using Leica Application Suite X (LAS X) software. The migratory and invasive cells, which migrated through the 5-μm-sized pores, were counted using Fiji analysis software (https://fiji.sc/).

### Immunofluorescence

For the evaluation of EMT and activation of the STAT3 signaling pathway, CT-26 cells were plated on glass coverslips at a cell density of 5 × 10^4^ cells/coverslip in 12-well plates (total volume of 1 mL). After 24 h, cells were treated with CM/sf DMEM (ratio 1:1), 10 µM CER, or 10 µM PA for 48 h. Cells were fixed with 1% PFA in PBS and stored at 4 °C until use. For immunofluorescence, fixed specimens were washed with 0.05% Tween-20 (Sigma-Aldrich,) in PBS for 5 min, followed by permeabilization with 0.1% Triton X-100 (Sigma-Aldrich) in PBS for 10 min. Next, cells were washed with 0.05% Tween-20 in PBS for 5 min and incubated in blocking solution containing 0.1% Triton X-100 and 5% normal goat serum (Dako, Glostrup, Denmark) in PBS for 30 min. The specimens were incubated with the primary antibodies in blocking solution at 4 °C overnight. The primary antibodies used were anti-Vimentin rabbit (1:100), anti-E-cadherin rabbit (1:100), and anti-STAT3 mouse (1:200). After washing, the specimens were incubated in blocking solution for 10 min followed by incubation with goat anti-mouse or goat anti-rabbit Alexa® Fluor 555-conjugated secondary antibodies in blocking solution (1:300) at room temperature for 60 min. DAPI (Life Technologies) in PBS (1:1000) was used for nuclear staining. Pertinent positive and negative controls were included in each batch of samples. Specimens were examined with a Leica DM5500 B fluorescence microscope, equipped with a Leica DFC365 FX digital camera. Digital images were acquired and stored using Leica Application Suite X (LAS X) software. Negative controls and treated groups were included in each batch of samples. In all groups, the cell reactivity was assessed by computerized densitometric analysis of the captured digital images with the aforementioned immunofluorescence microscope cited above. Average densitometric values from cell nucleus and cytoplasm were calculated in ImageJ software (https://rsb.info.nih.gov/ij/). Contrast index measurements were obtained from the formula [(selected area × 100)/total area] after removal of background in regions of interest (three samples per group).

### Western blot assay

To detect STAT3 and β-actin proteins, MC-38 cells were plated on dish at cell density of 2 × 10^6^ cells (total volume of 6 mL). After 24 h, cells were treated with CM/sf DMEM (ratio 1:1), 2.5 µM CER, or 2.5 µM PA for 48 h. To determine the total protein concentration of STAT3 and β-actin, the BCA protein assay was used (Thermo Fisher). Then, capillary electrophoresis was performed using the Protein Simple Wes according to the manufacturer’s instructions. Briefly, samples (cells lysates) were lysed with RIPA buffer containing 150 mM of sodium chloride, 1.0% NP-40, 0.5% sodium deoxycholate, 0.1% SDS, 50 mM of Tris, and a pH of 8.0 as well as protease inhibitor cocktail (Sigma-Aldrich),1 pill each for 10 mL of RIPA buffer. 2 μg/μL of lysed proteins were then mixed with the provided SDS/DTT mix, boiled at 95 °C for 5 min and loaded into prefilled microwell plate. Primaries antibodies, blocking buffer, luminol/peroxidase, HRP streptavidin, and secondaries anti-mouse and anti-rabbit antibodies provided by manufacturer (anti mouse and anti-rabbit detection module, protein simple) were then subsequently loaded into microplate and spin for 5 min at 300× g. The plate was then placed into the instrument for electrophoretic separation using 25-capillary cartridge for 12–230 kDa protein separation (SM-W004). Anti-STAT3 (Thermo Fisher, 1:50) and anti β-actin (BioLegend, 1:20) antibodies were used as primary antibodies for the assay. Chemiluminiscent bands were digitally generated and analyzed using the Compass software (ProteinSimple).

### Flow cytometry

EMT was examined with the two-chamber assay using a transwell co-culture system (Corning). RAW 264.7 cells (1 × 10^4^ cells) were seeded into the upper chamber of a transwell culture plate (insert) and MC-38 cells (5 × 10^4^ cells) were seeded on bottom of 24 well plate and incubated with DMEM medium containing 10% FBS for 24hrs. M2-TAM cells were incubated with sf DMEM and treated with 2.5 µM CER or 2.5 µM PA for 48 h. After incubation and treatment with 2.5 µM Cer or PA for 48 h, MC38 cells (1.5 × 10^4^ cells/well in a 12-well plate) were collected, washed with PBS, and incubated with 0.5% BSA in PBS for 45 min. To evaluate whether EMT was downregulated in cells by CER or PA, MC38 cells were labeled with E-cadherin-monoclonal antibody (DECMA-1), PerCP (1:1000) (eBioscience), or Vimentin monoclonal antibody (V9) FiTC (1:1000) (eBioscience). Following a final washing step, the cells were analyzed by flow cytometry.

### Statistical analysis

All experiments were performed in triplicate, and the significant differences between the groups were calculated using the analysis of variance and the Bonferroni's test, as indicated. A *p* < 0.05 was considered statistically significant.

## Results

### Low doses of Cer and PA did not affect cell viability

**Fig. 1:**
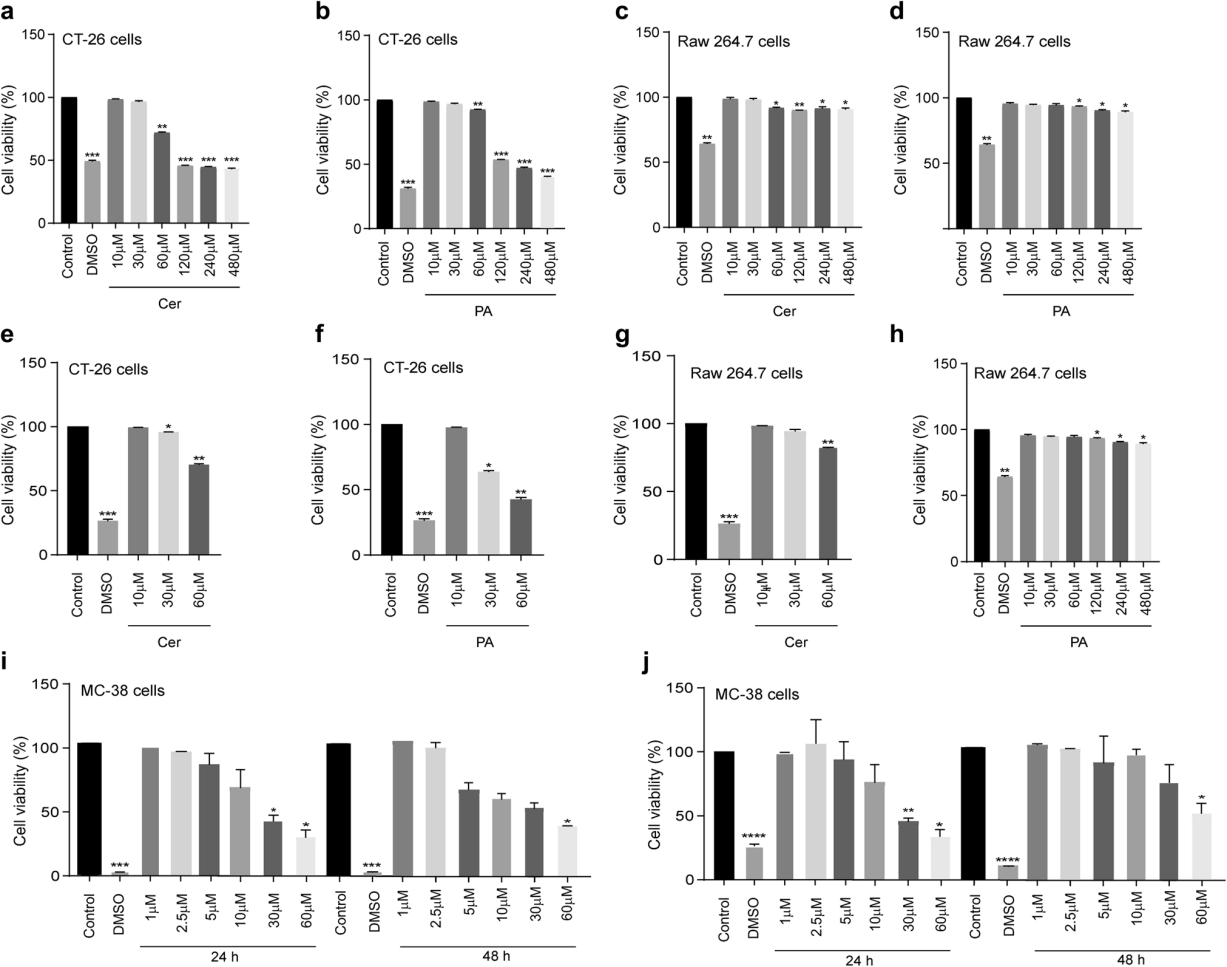
Cell viability of CT-26, RAW 264.7, and MC38 cells after treatment with Cer or PA for 24 h and 48 h. **a** CT-26 cell viability treated with increasing concentrations (10–480 μM) of **a** Cer or **b** PA after 24 h, assessed by MTS assay, or **e**, **f** after 48 h assessed by Hoechst labeling and flow cytometric analysis. **c**, **d** Cell viability of RAW 264.7 cells treated with increasing concentrations of Cer and PA, respectively, after 24 h, determined by MTS assay and **g, h** after 48hrs measured by flow cytometry. Cell viability of MC38 cells treated with increasing concentrations (1–60 μM) of **i** Cer or **j** PA for 24 h and 48 h. The data represent the mean ± SEM of 3–6 independent experiments. All *p* values were compared to control cells by analysis of variance and the Bonferroni's test, *p < 0.05; **p < 0.01 ***p < 0.001 versus control

First, we wanted to investigate whether PA and Cer modulate M2 TAM polarization and M2-promoted EMT of colon cancer cells. To ensure that changes in TAM polarization and migratory properties of colon cancer cells were caused by the influence of PA and Cer, and not by adverse cytotoxic effects, we first investigated whether Cer and PA applied in a concentration-dependent manner showed cytotoxicity towards CT-26 cells, RAW 264.7 cells and MC38 cells, determined by MTS assay (Fig. 1a–d, i and j). The viability of CT-26 and MC 38 cells decreased by 30% and 45%, respectively, when the cells were incubated for 24 h with 60 μM Cer, and the viability of CT-26 cells further decreased to 50%at 120 μM and higher doses, reaching a similar cytotoxic effect as DMSO (Fig. 1a). Similar to Cer, 60 μM PA reduced CT-26 cell viability by 10% and 55% in MC-38 cells after 24 h, and this increased to 50–60% when PA was applied at 120 μM or higher doses in CT-26 cells (Fig. 1b). After 48 h incubation with Cer or PA, also lower doses of 30 μM and 60 μM induced cell death in CT-26 cells, as determined by Hoechst labeling and flow cytometric analysis (Fig. 1e, f). In contrast, Cer applied at 60 μM to 240 μM reduced the viability of RAW 264.7 cells by only 10%, (Fig. 1c) and PA to a maximum of 20% after incubation with 120 μM to 240 μM for 24 h (Fig. 1d) and 48 h (Fig. 1g–h). When the cell viability of MC 38 cells is analyzed, it is perceived that Cer applied at 5 μM and 10 μM reduced the viability by 15–25% for 24 h (Fig. 1i) and 25–35% for 48 h (Fig. 1j). Whereas, PA applied at 5 μM and 10 μM reduced the viability by 10–25% for 24 h and 48 h. In conclusion, PA and Cer showed stronger cytotoxic effects on CT-26 and MC-38 cells than on RAW 264.7 cells. Thus, we performed all experiments at 2.5 μM or 10 μM Cer and PA. 2.5 μM concentration did not show cytotoxicity towards MC38 cells as well as 10 μM to CT-26 nor towards RAW 264.7 cells after 48 h incubation.

### Cer and PA attenuated M2 TAM polarization

**Fig. 2:**
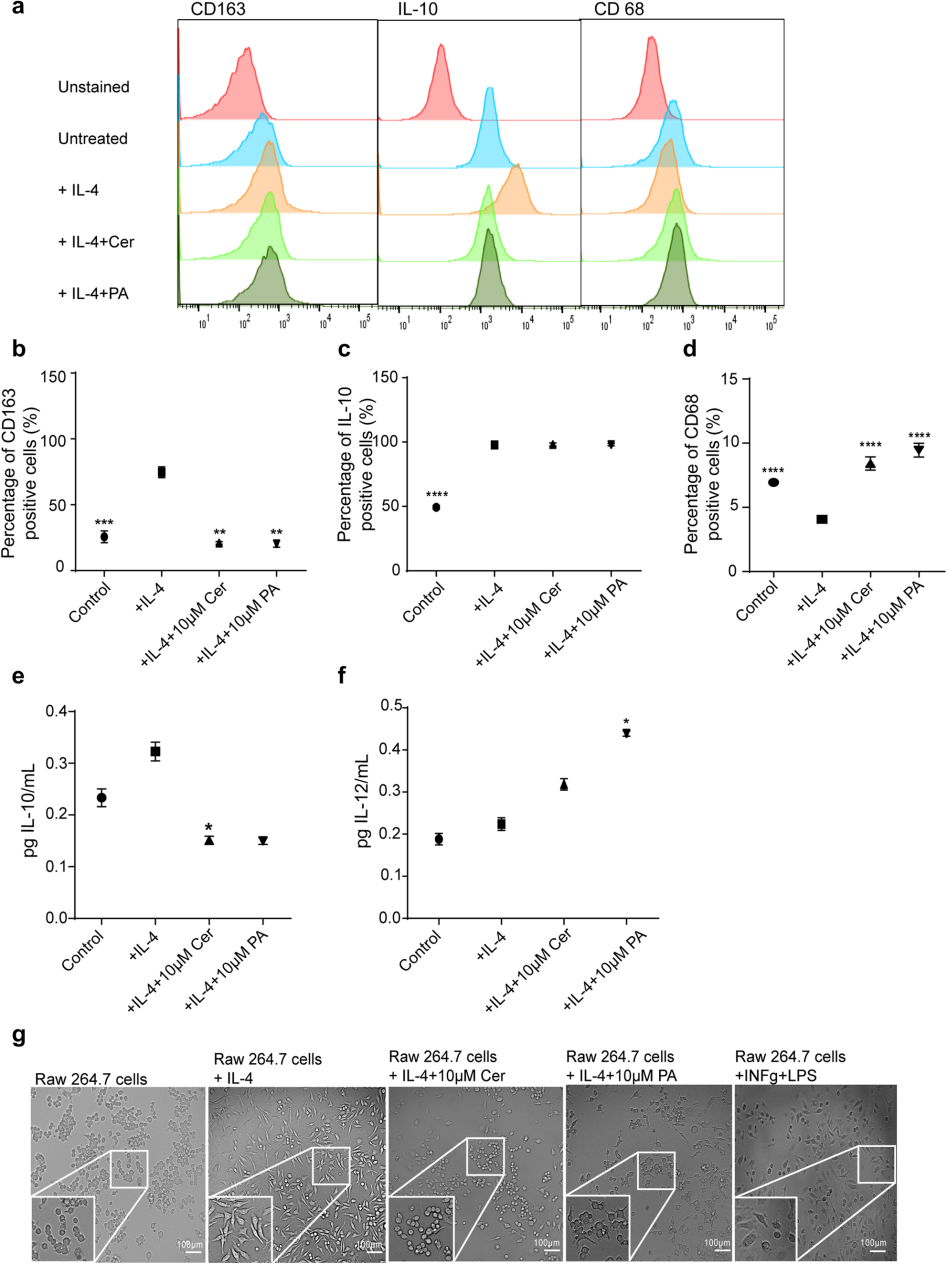
Cer and PA impair M2-TAM polarization. **a** Representative flow cytometry profiles of CD163, IL-10, and CD68 expression on RAW 264.7 control cells and upon treatment with IL-4, or IL-4 and Cer, or PA. **b** Percentage of positive cells of the M2 markers CD163 and **c** IL-10, and **d** the M1 marker CD68 on IL-4-polarized RAW 264.7 cells, in the presence and absence of 10 μM Cer or 10 μM PA during the 48 h polarization cell culture. ELISA analysis of the M2- and M1-related cytokines **e** IL-10 and **f** IL-12, respectively, from cell culture supernatants of IL-4-polarized RAW 264.7 cells, in the presence and absence of 10 μM Cer or 10 μM PA. All *p* values were compared to RAW 264.7cells + IL-4, ****p < 0.001 versus. The data represent the mean ± SEM of 3–6 independent experiments. All *p* values were compared to control cells by analysis of variance and the Bonferroni's test. **g** Representative phase-contrast images of control and IL-4 polarized RAW 264.7 cells, in the absence or presence of 10 μM Cer or 10 μM PA

To assess whether Cer and PA can modify the polarization of TAMs, we polarized RAW 264.7 macrophages towards M2-TAMs in the presence of IL-4 for 48 h and in the absence or presence of 10 μM Cer or 10 μM PA. To characterize these macrophages, the expression of M1- and M2-related markers in IL-4-stimulated RAW 264.7 cells were analyzed by flow cytometry. Flow cytometric analysis showed that expression of the M2-markers IL-10 and CD163 were significantly higher than in control RAW 264.7cells (Fig. 2a–c, p = 0.001 and *p* < 0.0001, respectively), demonstrating that IL-4 successfully induced the alteration of classical macrophages to M2-polarized TAMs. After treatment with 10 μM Cer and 10 μM PA, the expression of CD163, but not IL-10, significantly decreased (Fig. 2a–c, p < 0.001). In line with these findings, expression of the M1-related marker CD68 was reduced in M2-polarized TAMs (Fig. 2a, d) and the expression was significantly increased after addition of 10 μM Cer or 10 μM PA during the M2 polarization (*p* < 0.001 and *p* < 0.005, respectively).

To further characterize these macrophages, the cell culture supernatant was collected and the levels of M2- and M1-related cytokines IL-10 and IL-12, respectively, were measured by ELISA (Fig. 2e, f). Compared with control RAW 264.7cells, M2-polarized TAMs secreted significantly increased levels of IL-10 (Fig. 2e, *p* < 0.001). After treatment with 10 μM Cer and 10 μM PA, the levels of IL-10 decreased (both *p* < 0.005). In contrast, IL-12 was expressed at low levels in M2-polarized TAMs (Fig. 2f), but the levels doubled after treatment with 10 μM Cer and 10 μM PA (both *p* < 0.005).

M1- and M2-polarized macrophages are morphologically distinct, as observed using a phase-contrast microscope (Fig. 2g). RAW 264.7cells were polarized in the presence of LPS and IFN-y towards M1-TAMs that showed a flattened, “fried egg” morphology and abundance of vesicles compared to control RAW 264.7cells (Fig. 2g). In contrast, M2-polarized TAMs could be easily distinguished under the microscope. They were more elongated than the other two populations, with spindle-like cytoplasmic projections at the poles of the cells. After incubation with IL-4 and treatment with Cer and PA, the cells partially lost the morphological features of M2-TAMs and became more reminiscent of M1 and naïve RAW 264.7 cells (Fig. 2g).

### IL-10 showed pro-proliferative properties in colorectal cancer cells

**Fig. 3:**
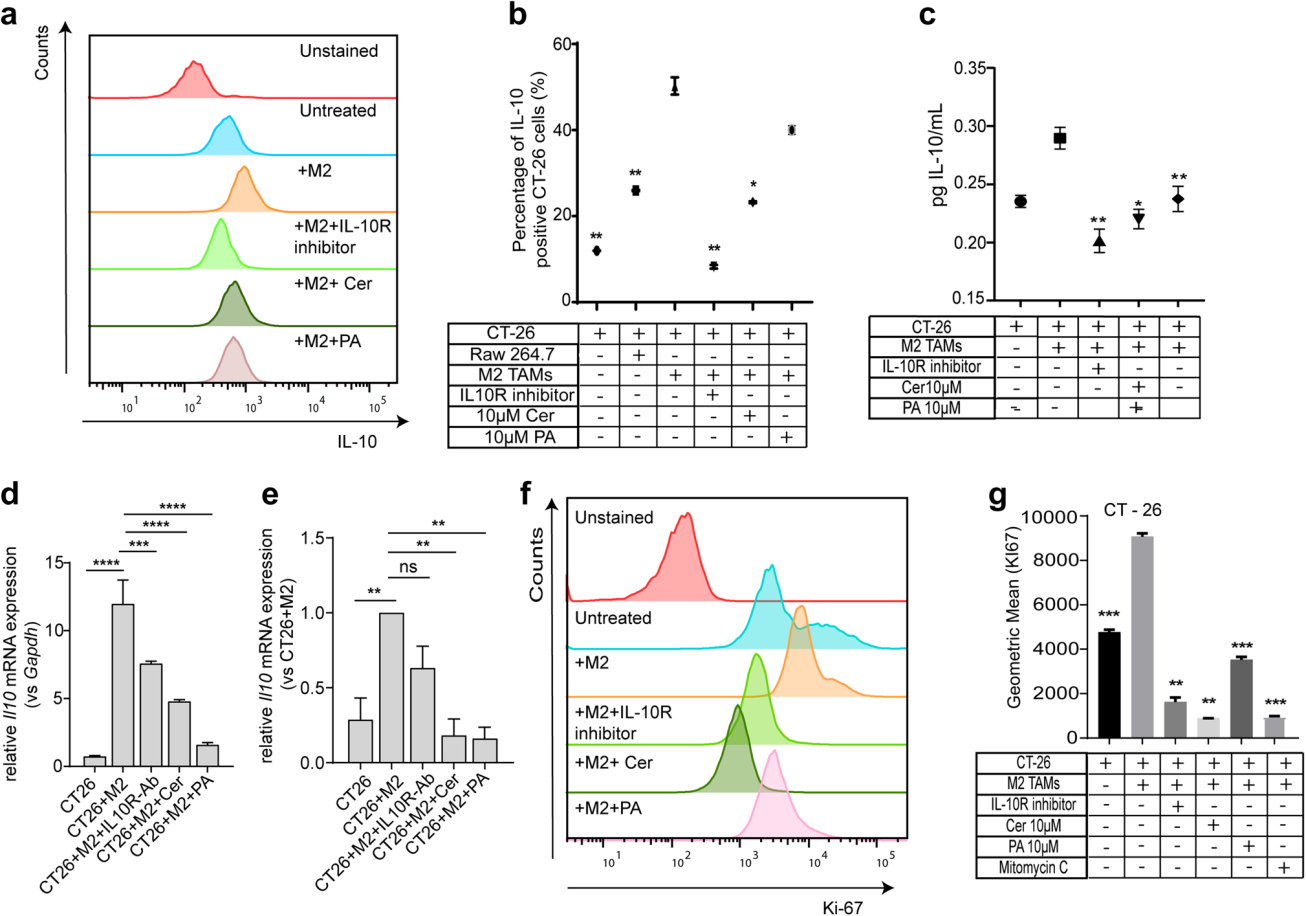
CT-26 cells indirectly co-cultured with PA- or Cer-treated M2-TAMs in a transwell system showed decreased expression of IL-10 and cell proliferation. **a** Representative flow cytometry profiles of IL-10 expression on control CT-26 cells and upon co-culture with IL-4, or IL-4 and 10 μM Cer, or 10 μM PA-treated RAW 264.7 cells. **b** Flow cytometric analysis of the mean fluorescent intensity of the M2 marker IL-10 in control CT-26 cells and upon co-culture with IL-4, IL-4 and Cer, or PA-treated RAW 264.7 cells. **c** ELISA analysis of the M2-related cytokine IL-10 in control CT-26 cells and upon co-culture with IL-4, IL-4 and Cer, or PA-treated RAW 264.7 cells. **d** Representative mRNA analysis of IL-10 transcription in CT-26 cells and upon co-culture with IL-4, IL-4 and Cer, or PA-treated RAW 264.7 cells, by real-time qPCR. Data are shown relative to *Gapdh* mRNA expression. **e** Normalized IL-10 mRNA expression in CT-26 cells. Changes in IL-10 expression are displayed as relative to CT-26 cells co-cultured with IL-4-treated RAW 264.7 cells. The data represent the mean ± SEM of 3–6 independent experiments. **f** Representative flow cytometry profiles and **g** quantification of the mean fluorescent intensity of Ki-67 expression in control CT-26 cells and upon co-culture with IL-4, IL-4 and Cer, or PA-treated RAW 264.7 cells. All *p* values were compared to CT-26 cells co-cultured with IL-4-treated RAW 264 cells by analysis of variance and the Bonferroni's test*p < 0.05; **p < 0.01; ***p < 0.001

IL-10 was highly upregulated in the CM of M2-polarized TAMs and expression was suppressed by the addition of Cer and PA (Fig. 2e). Next, we wondered whether M2-polarized TAMs and colorectal cancer cells could act in concert to establish an immunosuppressive milieu in the tumor microenvironment, and whether this interplay could be blocked by Cer and PA. To this end, CT-26 cells were indirectly co-cultured with PA or Cer-treated M2-TAMs in a transwell system, in the presence of an IL-10 receptor-blocking antibody. After 24 h, the expression of intracellular IL-10 was analyzed in CT-26 cells by flow cytometry (Fig. 3a, b). Non-treated CT-26 cells express IL-10, but upon co-culture with M2-TAMs, the levels of intracellular IL-10 in CT-26 cells significantly increased, and this was blocked by the addition of IL-10 receptor (IL-10R)-blocking antibody. Treatment of M2-TAMs with Cer significantly decreased IL-10 levels in CT-26, and there was a trend towards reduced expression after co-culture with PA-treated M2-TAMs (Fig. 3a, b). In line with these results, CT-26 secretes basal levels of IL-10, as determined by ELISA (Fig. 3c). The levels increased upon co-culture with M2-TAMs, and decreased below the basal expression level upon blocking of IL-10R, suggesting that IL-10 expression is partially regulated by a positive-feedback signaling via IL-10R. When co-cultured with PA or Cer-treated TAMs, the levels of IL-10 in the co-culture supernatant significantly decreased, compared to co-culture with non-treated M2-TAMs (Fig. 3c). To determine the changes in endogenous levels of IL-10 upon treatment with PA- or Cer-treated M2-TAMs inCT-26 cells, we analyzed the IL-10 mRNA expression in CT-26 cells indirectly co-cultured with M2-TAMs (Fig. 3d, e). In line with ELISA data, endogenous mRNA expression of IL-10 in CT-26 cells increased upon co-culture with M2-TAMs and was at least threefold reduced when M2-TAMs were pre-treated with PA or Cer. In summary, these results suggest that PA and Cer not only suppress IL-10 secretion by M2-TAMs, but also impact on IL-10 mRNA expression in colorectal cancer cells upon indirect co-culture, likely by a lack of positive-feedback signaling via IL10R.

Next we wondered whether reduced levels of IL-10 in CT-26 cells after co-culture with PA or Cer-treated M2-TAMs could influence cellular functions, such as cell proliferation. Thus, we assessed the percentage of proliferating cells by Ki-67 labeling. CT-26 cells were indirectly co-cultured with PA- or Cer-treated M2-polarized TAMs in a transwell system. After 24 h, CT-26 cells were analyzed by flow cytometry (Fig. 3f, g). When CT-26 cells were indirectly co-cultured with M2-polarized RAW cells, the expression of the proliferation marker Ki-67 doubled, and this effect was mediated by IL-10, as blocking of IL-10R reduced the expression levels of Ki-67 below the basal levels measured in CT-26 cells cultured in the absence of M2-TAMs. Interestingly, Cer was as potent in inhibiting proliferation as the IL-10R blocking antibody or mitomycin C, a proliferation inhibitor. PA also decreased the proliferation of CT-26 cells significantly.

In conclusion, IL-10 showed pro-proliferative effects on colorectal cancer cells, and this could be completely abolished by addition of Cer and PA to the co-culture.

### Cer and PA suppressed M2-TAM-induced migratory and invasive properties in CT-26 and MC-38 cells.

**Fig. 4:**
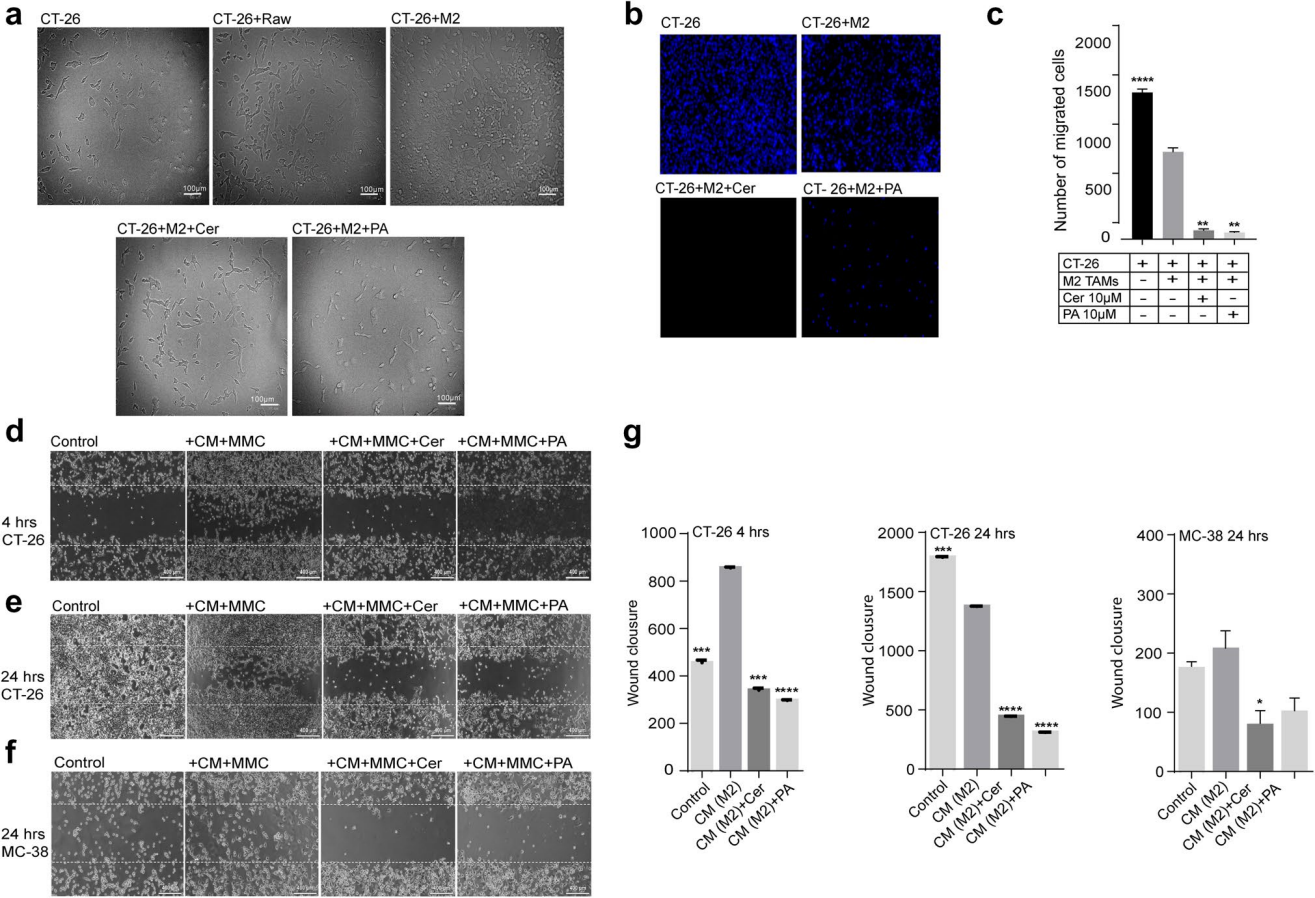
Cer and PA decreased the mesenchymal phenotype of colorectal cancer cells upon co-culture with M2-polarized TAMs or M2-conditional medium. **a** Phase-contrast images of CT-26 cells indirectly co-cultured with M2-polarized TAMs in a transwell system for 24 h, in the presence or absence of 10 μM Cer or 10 μM PA. Scale bar = 100 µm. **b** Immunofluorescence microscopy images of transmigrated CT-26 cells. CT-26 cells were indirectly co-cultured with M2-polarized TAMs in a transwell system for 24 h, in the presence or absence of 10 μM Cer or 10 μM PA. The cell nuclei of transmigrated cells at the bottom of the transwell inset were stained with DAPI (blue) and **c** quantified using Fiji image analysis software (see Materials and methods). Scale bar = 25 µm. **d-f** Migratory properties of CT-26 and MC-38 cells after co-culture with CM of M2-TAMs were assessed in a wound-healing assay. Phase-contrast images of CT-26 cells in a wound-healing assay after 4 and 24 h. CT-26 cells were cultured in the presence of CM of M2-TAMs, with CM of M2-TAMs treated with 10 μM Cer, or 10 μM PA and the proliferation-inhibitor Mitomycin C. The dotted lines represent the wound (scratched) area. Scale bar = 40 µm. MMC = Mitomycin C. Quantification of migrated colorectal cancer cells into the wound area (area between the dotted lines) after **g** 4 and 24 h. All *p* values were compared to CT-26 and MC-38 cells co-cultured with CM of IL-4-treated RAW 264 cells by analysis of variance and the Bonferroni's test. **p < 0.01; ***p < 0.001, ***p < 0.0001 versus CT-26 and MC38 cells in co-culture with M2-TAMs or M2-TAM CM

TAMs and cancer cells closely interact in the tumor microenvironment and M2-TAMs have been shown to promote EMT of cancer cells. Here, we investigated whether Cer and PA could affect M2-TAM-induced EMT of CT-26 cells. To this end, CT-26 cells were either cultured alone, or indirectly co-cultured with M2-polarized TAMs in a transwell system. Changes in cell morphology, indicative of an epithelial or mesenchymal phenotype, were examined using a phase-contrast microscope (Fig. 4a). CT-26 cells are adherent cells with a fibroblast-like morphology (Fig. 4a). When indirectly co-cultured with M2-polarized TAMs, and not with naïve macrophages, CT-26-cells showed numerous long membrane protrusions that radiated outwards from the cell body, reminiscent of a mesenchymal migratory phenotype (Fig. 4a). However, when CT-26 cells were co-cultured with M2-polarized TAMs in the presence of 10 μM Cer or 10 μM PA, cell protrusions were visually decreased and the mesenchymal phenotype was less prominent (Fig. 4a).

Along with the ability to undergo EMT, tumor cells exhibit increased mobility. To investigate whether M2-polarized TAMs could directly impact on the mobility of colorectal cancer cells, we measured the number of CT-26 to migrate through the pores of a transwell system in presence and absence of M2-polarized TAMs. CT-26 cells cultured in the inset of a transwell were either cultured alone, or indirectly co-cultured with M2-polarized TAMs at the bottom of the transwell system. After 24 h, the inset with CT-26 cells was separated and non-transmigrated cells were physically removed (see Materials and methods). The transmigrated cells were stained with DAPI and analyzed by fluorescent microscopy (Fig. 4b, c). M2-polarized TAMs stimulated migration of CT-26, compared to non-treated cells (Fig. 4b, c, *p* < 0.0001), and this effect could be blocked by the addition of Cer (*p* < 0.003) and PA (*p* < 0.002) during the co-culture (Fig. 4b, c). These results suggest that Cer and PA inhibit the process by which M2-TAMs promote the migration of CT-26-cells.

Next, we wondered whether Cer and PA inhibit migration of CT-26 and MC-38 cells by directly impacting on cancer cells or via M2-polarized TAMs. To this end, we quantified the migratory properties of CT-26 and MC-38 cells in a wound-healing assay. Cancer cell lines were treated with supernatant (CM) of M2-TAMs, which were cultured in the presence or absence of PA and Cer. To ensure that we measured cell migration into the wound area, and not passive cell movement due to cell proliferation, we treated CT-26 and MC-38 cells during this experiment with mitomycin C, an inhibitor of cellular proliferation (Fig. 4d–f;). After 4 (*p* < 0.0001) and 24 h (p < 0.0001), we observed migration of CT-26 cells incubated with CM into the wound area, while after 24 h, the control cells without addition of mitomycin (but CM) had already closed the wound area (Fig. 4e, g). In contrast, when CT-26 and MC-38 cells were treated with mitomycin C, and CM from M2-TAMs were treated with Cer or PA, significantly fewer cells migrated into the wound area after 4 and 24 h (Fig. 4d–g, Cer, *p* < 0.0001 and PA, *p* < 0.0001 4 h and 24 h to CT-26 cells; Cer, *p* < 0.05 and PA, *p* > 0.05 24 h to MC-38 cells).

In conclusion, these data indicate that M2-TAMs promote EMT and cell mobility of colorectal cancer cells, and these processes were suppressed by Cer and PA. Additionally, our data demonstrate that the inhibition of cancer cell migration was conferred to factors present in the CM of M2-TAMs.

### Cer and PA decreased the mesenchymal phenotype in colorectal cancer cells

**Fig. 5:**
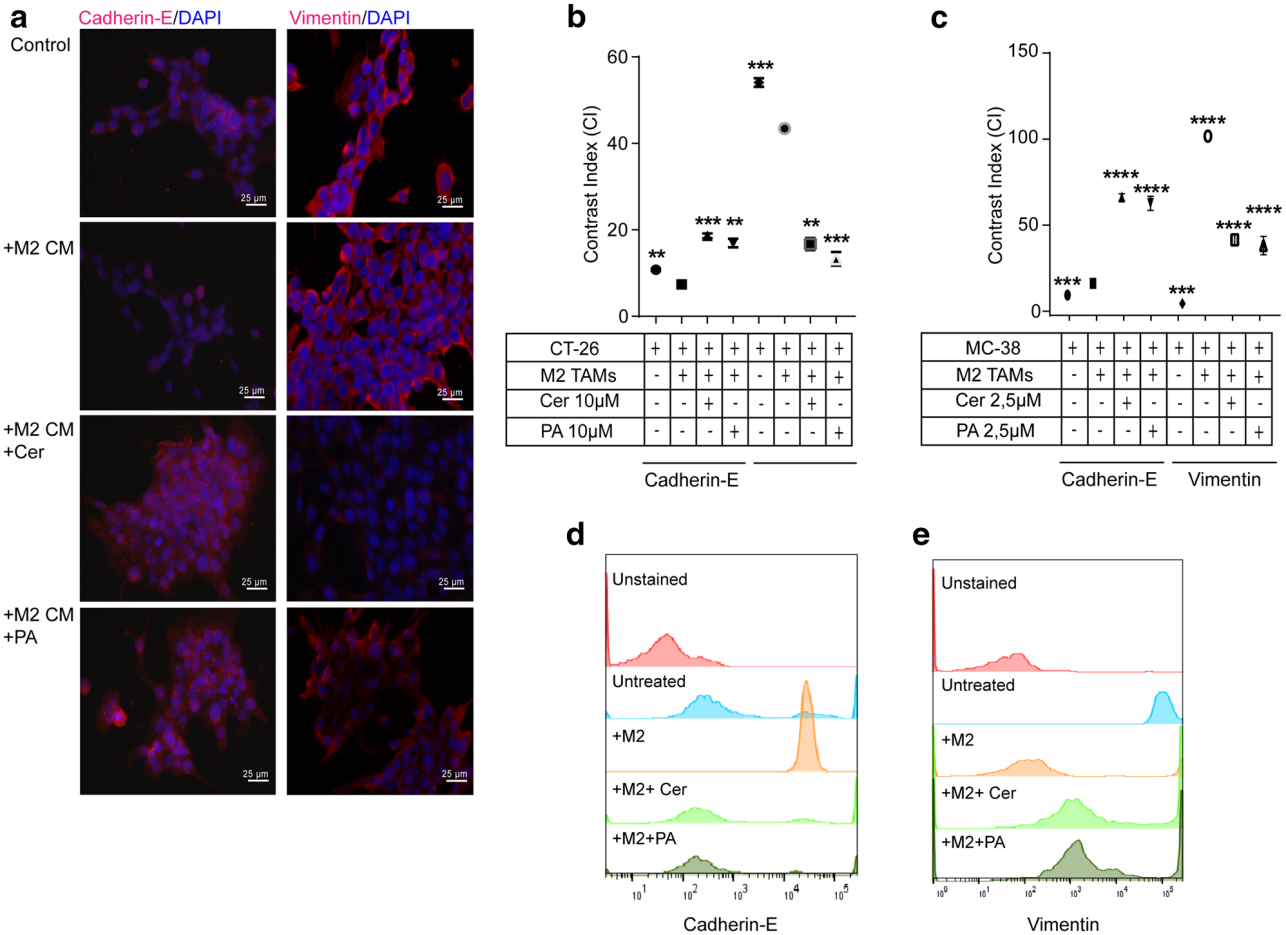
Co-culture of CT-26 cells with M2-polarized tumor-associated macrophages (TAMs) increased the mesenchymal phenotype in colorectal cancer cells. **a** CT-26 cells were indirectly co-cultured with CM of PA- or Cer-treated (10 μM each) M2-TAMs for 48 h and analyzed by fluorescent microscopy for Cadherin-E (left, purple) and Vimentin (right, purple) expression. The cell nuclei were stained with DAPI (blue). Scale bar = 25 µm. **b** Quantification of the fluorescent intensity of the Cadherin-E and Vimentin labeling in CT-26 cells upon co-culture with CM of PA- or Cer-treated M2-TAMs. **c** MC-38 cells were directly co-cultured with M2-TAMs treated that were with PA or Cer (2.5 μM each) for 48 h and analyzed by flow cytometer for **d** Vimentin and** e** Cadherin-E expression. All *p* values were compared to CT-26 cells co-cultured with CM of IL-4-treated RAW 264 as well as compared to MC-38 cells directly co-cultured with IL-4-treated RAW 264 by analysis of variance and Bonferroni's test **p < 0.01, ***p < 0.001 versus M2-TAM CM or M2-TAM

Tumor cells undergoing EMT show characteristic changes in phenotype, which are closely related to their increased migratory properties. Thus, we wondered whether PA and Cer suppress cell mobility by altering the genetic program in colorectal cancer cells leading to up or downregulation of EHT markers. To this end, CT-26 cells and MC-38 cells were cultured in the presence M2-TAM CM or M2-TAM and the expression of the epithelial marker E-cadherin and the mesenchymal marker Vimentin were analyzed by immunofluorescent microscopy (Fig. 5a, b) and flow cytometry (Fig. 5c–e). CT-26 and MC38 cells alone, or cultured in the presence of M2-TAM CM or M2-TAM, respectively, showed low expression of E-cadherin. However, when CT-26 and MC-38cells were cultured in the presence of CM from PA- or Cer-treated M2-TAMs and PA- or Cer- treated M2, respectively, the expression of the E-cadherin significantly increased (*p* < 0.0001 and *p* < 0.001, respectively) in CT-26 cells and in MC-38 cells (Fig. 5a, b; *p* < 0.0001 and Fig. 5c, e; *p* < 0.0001, respectively). In contrary, the expression of Vimentin was high on non-treated CT-26 and MC-38 cells, indicating that both of them possess a mesenchymal phenotype. Upon addition of CM from M2-TAMs in CT-26 cells or co-cultured MC-38 with M2-TAMs, Vimentin expression slightly but significantly increased (*p* < 0.0001; *p* < 0.0001, respectively). However, the expression decreased in both of cells when M2-TAMs were pre-treated with PA or Cer (Fig. 5a, b, *p* < 0.0001 and *p* < 0.001, respectively; Fig. 5c, d, *p* < 0.0001 and *p* < 0.0001, respectively). Our data indicate that Cer and PA suppress cell mobility by altering the genetic program and, consequently, EMT marker expression in CT-26 and MC-38 cells, and this effect was mediated by the CM of M2-polarized TAMs pre-treated with PA or Cer and TAMs pre-treated with PA or Cer, respectively.

### STAT-3 and NF-κB signaling play a key role in M2-TAM-promoted EMT in colorectal cancer cells

**Fig. 6:**
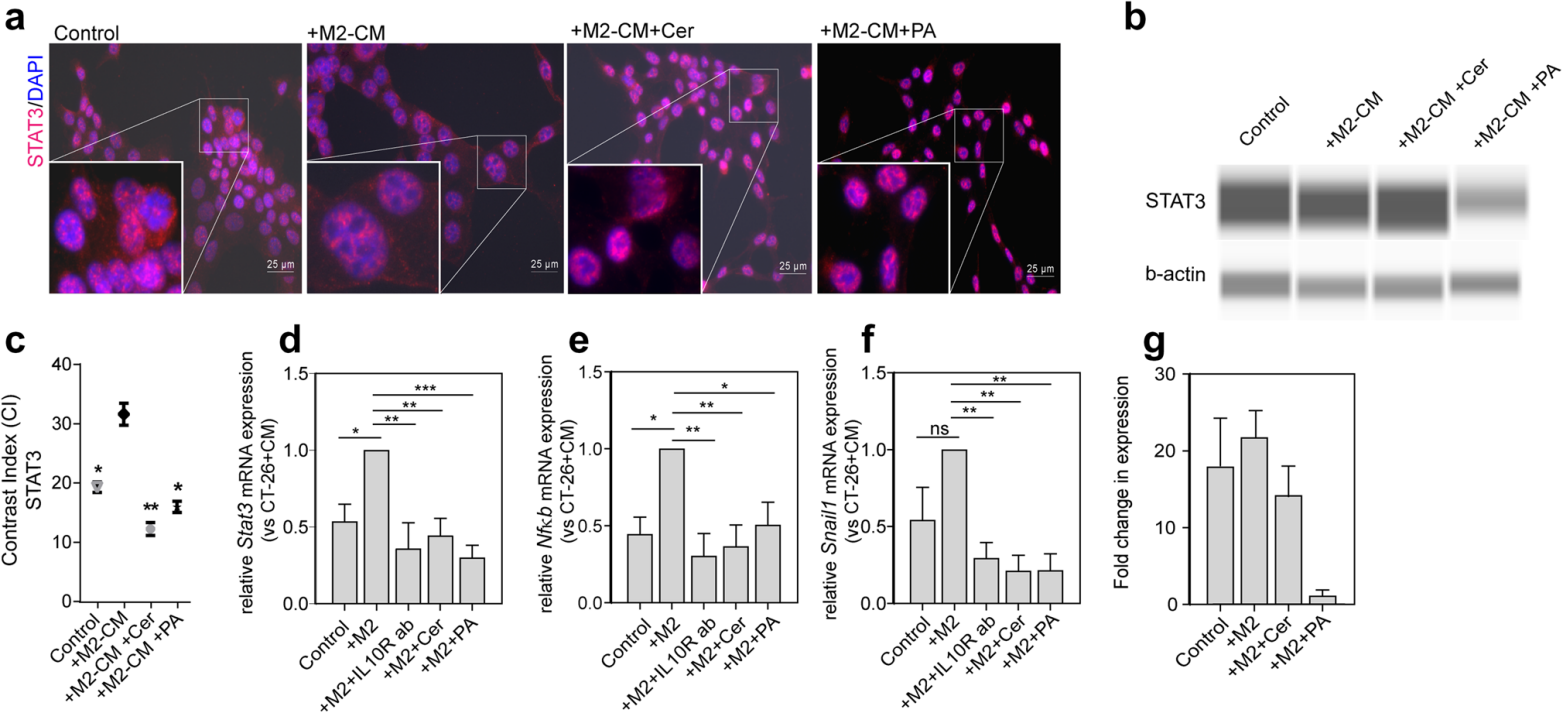
Co-culture of CT-26 cells with CM of M2-TAMs increased the expression of STAT-3 and NF-κB in colorectal cancer cells. **a** CT-26 cancer cells were indirectly co-cultured with CM from PA- or Cer-treated M2-TAMs. After 48 h, STAT-3 protein expression (purple) in CT-26 cells was analyzed by immunofluorescent microscopy. The cell nuclei were stained with DAPI (blue), **c** quantification of the fluorescent intensity of the STAT-3 labeling. **b, g** STAT-3 protein levels were determined in MC-38 cells co-cultured with M2-TAM and treated with PA and Cer (2.5 μM each) by Western blot analysis with β-actin as a loading control. Three independent experiments were performed for quantification (mean ± standard deviation) (lower panel). Normalized **d***Stat3*, **e***Nfkb*, and **f***Snail1* mRNA expression in CT-26 cells. Changes in mRNA expression are displayed as relative to CT-26 cells co-cultured with IL-4-treated M2-polarized RAW 264.7 cells. The data represent the mean ± SEM of 3–6 independent experiments. All *p* values were compared to CT-26 cells co-cultured with IL-4-treated RAW 264 cells by one-way ANOVA with Dunnett’s multiple comparison test. **p < 0.01, ***p < 0.001 versus M2-TAM

Previous studies implicated that STAT-3 and NF-κB modulate colorectal cancer progression and metastasis [19]. In the present study, we examined whether STAT-3 and NF-κB play a role in the M2-TAM-induced EMT of CT-26 cells. First, we analyzed the protein levels of STAT-3 in CT-26 and MC-38 cells by immunofluorescent microscopy and Western blot, respectively (Fig. 6a, c, b, and g). STAT-3 was highly expressed in the cytoplasm and to a lesser extent in the nucleus of control CT-26 cells and CT-26 cells treated with CM of M2-TAMs. When M2-TAMs were pre-treated with PA or Cer, the expression of STAT-3 in CT-26 cells, cultured in the presence of CM, was significantly reduced (Fig. 6a, c). On the other hand, the total protein expression of STAT-3 was completely reduced in MC-38 cells pre-treated with PA, but not with Cer (Fig. 6b, g). The immunofluorescence data was confirmed by RT-qPCR data (Fig. 6d). mRNA expression of STAT-3 in CT-26 increased, when the cells were treated with CM of M2-TAMs, and the expression decreased when M2-TAMs were pre-treated with PA or Cer. Next to STAT3, we analyzed the mRNA expression of NF-κB and its downstream target and inhibitor of E-cadherin, SNAI1. Similar to STAT-3, NF-κB (Fig. 6e) and SNAI1 (Fig. 6f) mRNA expression in CT-26 cells was upregulated upon culture with CM of M2-TAMs. Upon pre-treatment of M2-TAMs with PA or Cer, the levels of NF-κB and SNAI1 mRNA significantly decreased (Fig. 6e, f). In conclusion, our data indicate that STAT-3 and NF-κB are involved in the M2-TAM-promoted EMT process of colorectal cancer cells and that PA and Cer decrease the mRNA expression of STAT-3, NF-κB, and SNAI1.

## Discussion

The present study reveals that Cer and PA exert anti-tumor effects by blocking polarization of M2-polarized TAMs and ,consequently, EMT of colorectal cancer cells. First, we showed that Cer and PA treatment attenuated macrophage polarization towards the M2 phenotype by suppressing the expression of the M2-related cytokine IL-10. Secondly, we demonstrated that IL-10 produced by M2-TAMs induced EMT in colorectal cancer cells and that Cer and PA blocked this process by inhibition of IL-10 expression and the EMT-related signaling molecules STAT3, Snail, and NF-κB in colorectal cancer cells.

Immune cells participate in several processes in the tumor microenvironment and have been associated with tumor progression. Macrophages in the tumor microenvironment are mainly M2-polarized TAMs and release anti-inflammatory cytokines (e.g., IL-1, TNF-a, IL-10) [4, 20]. While in healthy individuals, M2-alternative-activated macrophages are involved in tissue repair and remodeling; they may participate in all aspects of tumor cell invasion and metastasis in the tumor [5, 39]. Thus, macrophage function and phenotype are highly dependent on their microenvironment [40]. Lipids (cellular or dietary) and alterations in lipid metabolism have long been identified as regulators of immune cell function and macrophage polarization [27, 41–44]. Consistent with previous reports, our data demonstrate that PA and Cer attenuate the M2-phenotype in macrophages. PA is a major saturated fatty acid and has been shown to stimulate pro-inflammatory cytokine expression by macrophages in vitro [28, 45] and in pathologic processes like obesity. PA can directly bind to TLRs, or together with LPS they induce macrophages to express inflammatory cytokines [28, 29]. In addition, macrophage can accumulate lipids intracellularly via scavenger receptors, and cause endoplasmatic stress and mitochondrial dysfunction, which drives the M1 phenotype and inflammatory response [26]. Cer, a sphingolipid metabolite, plays an important role in many pathobiological processes, such as growth arrest, apoptosis, cellular signaling adhesion, and trafficking of immune cells [41, 46]. Cer is also a powerful tumor suppressor, and has recently been shown to induce anti-tumor immunity by targeting M2-TAMs through inhibition of ROS signaling [36]. Albeit being different lipid species, PA and Cer block M2-TAM polarization. Previous studies demonstrated that Cer is synthesized upon PA-amplified LPS-signaling, and stimulates the transcription of pro-inflammatory cytokines [43, 47]. Thus, it is likely that addition of Cer, a downstream signaling mediator of PA-mediated TLR signaling, directly stimulates the same pro-inflammatory response as PA.

IL-10 is a pleiotropic cytokine produced by myeloid cells and lymphocytes that display both immunoregulatory and immunostimulatory effects [48]. IL-10 has also been shown to inhibit the production of IL-12, downregulate MHC-I expression, and reduce the Th1 response. It is well known that IL-10 produced by macrophages decreases the anti-tumor T cell response and facilitates tumor growth [49]. We and others found PA and Cer, two chemically different lipids species, to inhibit IL-10 expression; however, the underlying mechanism is not fully understood. Lipids, such as PA and Cer are components of the plasma membrane. Changes in Cer levels in the plasma membrane of immune cells have been shown to affect plasma membrane composition and compartmentalization [41] and signaling pathways, such as PI3 kinase-Akt pathway [50]. Addition of PA and Cer might affect macrophage plasma membrane properties and thereby alter the signaling pathways underlying M2-TAM polarization, resulting in a different cytokine profile, marker expression, and morphology.

The main focus of this study was to investigate whether Cer and PA could block the M2-TAM-induced EMT of colorectal cancer cells. EMT has been implicated in the progression of colon cancer [51]; however, no studies have addressed whether lipids would interfere in the induction of EMT in colorectal cancer cells promoted by M2-polarized TAMs. While it is known that IL-6 promotes the EMT of cancer cells [52], our study showed that IL-10, secreted by M2-TAMs, induced EMT in colorectal cancer cells. Overexpression of IL-10 has been shown to confer tumor growth and is associated with poor prognosis in breast cancer, highlighting the tumorigenic properties of IL-10[17]. We further demonstrate that IL-10 stimulates EMT in colorectal cancer cells by activation of the STAT3- and NF-κB pathways and by promoting cancer cell proliferation. STAT3 expression and activity is dysregulated in many malignant cancers, including breast, skin, brain, and colon cancer [51]. STAT3 shuttles from the cytoplasm to the nucleus, where it binds via a coiled-coil domain to modulate important genes involved in invasion and apoptosis [53, 54]. Consistent with our findings, it has been shown that IL-10R signaling is involved in overstimulation of STAT3, enabling tumor cells to grow uncontrollably and to become resistant to the induction of apoptosis [16], confirming the link demonstrated here between IL-10 and STAT3. Thus, IL-10 promotes STAT3 activation, and persistent activation is significantly associated with poor prognosis and metastasis [52]. Other studies suggest that IL-10 stimulates the EMT process and increases proliferation in cancer cells via a STAT3-NF-κB-IL-10 signaling axis [19, 55]. In agreement, we detected an increase in NF-κB expression in CT-26 cells co-cultured with M2-TAM CM, and this could be blocked by PA and Cer. At the molecular level, it was reported that STAT3 can directly bind NF-κB and mediates its nuclear translocation and NF-κB target gene expression, thus showing that STAT3 plays a role in the constitutive activation of NF-κB [54]. Thus, PA and Cer likely reduce the levels of NF-κB by impacting on STAT3 levels and activity state.

STAT3 and its signal mediators are potential pharmacological and gene therapy targets and hold great promise for cancer therapy [56].

## Conclusions

Here, we show for the first time that PA and Cer are powerful inhibitors of the IL-10-STAT3-NF-κB signaling axis in a co-culture system consisting of M2-TAMs and colorectal cancer cells, mimicking the tumor microenvironment. Inhibition of IL-10 secretion by M2-TAMs resulted in impairment of EMT, migratory, and proliferative capacities of colorectal cancer cells. PA and Cer are non-toxic lipids and natural components of the plasma membrane, and delivered in vivo at significantly high concentrations, as recently demonstrated for Cer [36], and could be powerful anti-tumor agents.

## Abbreviations

Cer: Ceramide
PA: Palmitic acid
EMT: Epithelial–mesenchymal transition
M1: Macrophage 1
M2: Macrophage 2
TAMs: Tumor-associated macrophages
LPS: Lipopolysaccharide
IFN-γ: Interferon gamma
TNF-α: Tumor necrosis factor alpha
IL: Interleukin
MHC: Major histocompatibility complex
STAT3: Signal transducer and activator of transcription 3
E-cad: E-cadherin
CM: Conditioned medium
FBS: Fetal bovine serum

## References

[1] Bray F, Ferlay J, Soerjomataram I, Siegel RL, Torre LA, Jemal A (2018). Global cancer statistics 2018: GLOBOCAN estimates of incidence and mortality worldwide for 36 cancers in 185 countries. CA Cancer J Clin.

[2] Yona S, Kim KW, Wolf Y, Mildner A, Varol D, Breker M (2013). Fate mapping reveals origins and dynamics of monocytes and tissue macrophages under homeostasis. Immunity.

[3] Muraille E, Leo O, Moser M (2014). TH1/TH2 paradigm extended: macrophage polarization as an unappreciated pathogen-driven escape mechanism?. Front Immunol.

[4] Qian BZ, Pollard JW (2010). Macrophage diversity enhances tumor progression and metastasis. Cell.

[5] Salmaninejad A, Valilou SF, Soltani A, Ahmadi S, Abarghan YJ, Rosengren RJ (2019). Tumor-associated macrophages: role in cancer development and therapeutic implications. Cell Oncol.

[6] Sica A, Invernizzi P, Mantovani A (2014). Macrophage plasticity and polarization in liver homeostasis and pathology. Hepatology.

[7] Mulder R, Banete A, Seaver K, Basta S (2017). M(IL-4) tissue macrophages support efficient interferon-gamma production in antigen-specific CD8(+) T cells with reduced proliferative capacity. Front Immunol.

[8] Allavena P, Mantovani A (2012). Immunology in the clinic review series; focus on cancer: tumour-associated macrophages: undisputed stars of the inflammatory tumour microenvironment. Clin Exp Immunol.

[9] Mantovani A, Sica A, Sozzani S, Allavena P, Vecchi A, Locati M (2004). The chemokine system in diverse forms of macrophage activation and polarization. Trends Immunol.

[10] Lamouille S, Xu J, Derynck R (2014). Molecular mechanisms of epithelial–mesenchymal transition. Nat Rev Mol Cell Biol.

[11] Nijkamp MM, Span PN, Hoogsteen IJ, van der Kogel AJ, Kaanders JH, Bussink J (2011). Expression of E-cadherin and vimentin correlates with metastasis formation in head and neck squamous cell carcinoma patients. Radiother Oncol.

[12] Kalluri R, Weinberg RA (2009). The basics of epithelial–mesenchymal transition. J Clin Invest.

[13] Palma Cde S, Grassi ML, Thome CH, Ferreira GA, Albuquerque D, Pinto MT (2016). Proteomic analysis of epithelial to mesenchymal transition (EMT) reveals cross-talk between SNAIL and HDAC1 proteins in breast cancer cells. Mol Cell Proteomics.

[14] Dethlefsen C, Hojfeldt G, Hojman P (2013). The role of intratumoral and systemic IL-6 in breast cancer. Breast Cancer Res Treat.

[15] Bishop JL, Thaper D, Zoubeidi A (2014). The multifaceted roles of STAT3 signaling in the progression of prostate cancer. Cancers.

[16] Valle Oseguera CA, Spencer JV (2014). cmvIL-10 stimulates the invasive potential of MDA-MB-231 breast cancer cells. PLoS ONE.

[17] Liu J, Wang L, Gao W, Li L, Cui X, Yang H (2014). Inhibitory receptor immunoglobulin-like transcript 4 was highly expressed in primary ductal and lobular breast cancer and significantly correlated with IL-10. Diagn Pathol.

[18] Pahl HL (1999). Activators and target genes of Rel/NF-kappaB transcription factors. Oncogene.

[19] Grivennikov SI, Karin M (2010). Dangerous liaisons: STAT3 and NF-kappaB collaboration and crosstalk in cancer. Cytokine Growth Factor Rev.

[20] Grivennikov SI, Greten FR, Karin M (2010). Immunity, inflammation, and cancer. Cell.

[21] Yadav A, Kumar B, Datta J, Teknos TN, Kumar P (2011). IL-6 promotes head and neck tumor metastasis by inducing epithelial–mesenchymal transition via the JAK-STAT3-SNAIL signaling pathway. Mol Cancer Res.

[22] Condeelis J, Segall JE (2003). Intravital imaging of cell movement in tumours. Nat Rev Cancer.

[23] Genard G, Lucas S, Michiels C (2017). Reprogramming of tumor-associated macrophages with anticancer therapies: radiotherapy versus chemo- and immunotherapies. Front Immunol.

[24] Kalish SV, Lyamina SV, Usanova EA, Manukhina EB, Larionov NP, Malyshev IY (2015). Macrophages reprogrammed in vitro towards the M1 phenotype and activated with LPS extend lifespan of mice with ehrlich ascites carcinoma. Med Sci Monit Basic Res.

[25] Keklikoglou I, Cianciaruso C, Guc E, Squadrito ML, Spring LM, Tazzyman S (2019). Chemotherapy elicits pro-metastatic extracellular vesicles in breast cancer models. Nat Cell Biol.

[26] Remmerie A, Scott CL (2018). Macrophages and lipid metabolism. Cell Immunol.

[27] Talamonti E, Pauter AM, Asadi A, Fischer AW, Chiurchiu V, Jacobsson A (2017). Impairment of systemic DHA synthesis affects macrophage plasticity and polarization: implications for DHA supplementation during inflammation. Cell Mol Life Sci.

[28] Cullberg KB, Larsen JO, Pedersen SB, Richelsen B (2014). Effects of LPS and dietary free fatty acids on MCP-1 in 3T3-L1 adipocytes and macrophages in vitro. Nutr Diabetes.

[29] Lee JY, Sohn KH, Rhee SH, Hwang D (2001). Saturated fatty acids, but not unsaturated fatty acids, induce the expression of cyclooxygenase-2 mediated through Toll-like receptor 4. J Biol Chem.

[30] Zhou BR, Zhang JA, Zhang Q, Permatasari F, Xu Y, Wu D (2013). Palmitic acid induces production of proinflammatory cytokines interleukin-6, interleukin-1beta, and tumor necrosis factor-alpha via a NF-kappaB-dependent mechanism in HaCaT keratinocytes. Mediat Inflamm.

[31] Wang SW, Hojabrpour P, Zhang P, Kolesnick RN, Steinbrecher UP, Gomez-Munoz A (2015). Regulation of ceramide generation during macrophage apoptosis by ASMase and de novo synthesis. Biochim Biophys Acta.

[32] Spiegel S, Foster D, Kolesnick R (1996). Signal transduction through lipid second messengers. Curr Opin Cell Biol.

[33] Kolesnick R, Golde DW (1994). The sphingomyelin pathway in tumor necrosis factor and interleukin-1 signaling. Cell.

[34] Hannun YA (1996). Functions of ceramide in coordinating cellular responses to stress. Science.

[35] Sawai H, Okazaki T, Takeda Y, Tashima M, Sawada H, Okuma M (1997). Ceramide-induced translocation of protein kinase C-delta and -epsilon to the cytosol. Implications in apoptosis. J Biol Chem.

[36] Li G, Liu D, Kimchi ET, Kaifi JT, Qi X, Manjunath Y (2018). Nanoliposome C6-ceramide increases the anti-tumor immune response and slows growth of liver tumors in mice. Gastroenterology.

[37] Liu CY, Xu JY, Shi XY, Huang W, Ruan TY, Xie P (2013). M2-polarized tumor-associated macrophages promoted epithelial–mesenchymal transition in pancreatic cancer cells, partially through TLR4/IL-10 signaling pathway. Lab Investig.

[38] Tanaka K, Arao T, Maegawa M, Matsumoto K, Kaneda H, Kudo K (2009). SRPX2 is overexpressed in gastric cancer and promotes cellular migration and adhesion. Int J Cancer.

[39] Liu YC, Zou XB, Chai YF, Yao YM (2014). Macrophage polarization in inflammatory diseases. Int J Biol Sci.

[40] Lumeng CN, Bodzin JL, Saltiel AR (2007). Obesity induces a phenotypic switch in adipose tissue macrophage polarization. J Clin Investig.

[41] Eich C, Manzo C, de Keijzer S, Bakker GJ, Reinieren-Beeren I, Garcia-Parajo MF (2016). Changes in membrane sphingolipid composition modulate dynamics and adhesion of integrin nanoclusters. Sci Rep.

[42] Hubler MJ, Kennedy AJ (2016). Role of lipids in the metabolism and activation of immune cells. J Nutr Biochem.

[43] Jin J, Lu Z, Li Y, Cowart LA, Lopes-Virella MF, Huang Y (2018). Docosahexaenoic acid antagonizes the boosting effect of palmitic acid on LPS inflammatory signaling by inhibiting gene transcription and ceramide synthesis. PLoS ONE.

[44] Lochner M, Berod L, Sparwasser T (2015). Fatty acid metabolism in the regulation of T cell function. Trends Immunol.

[45] Jin J, Lu Z, Li Y, Ru JH, Lopes-Virella MF, Huang Y (2018). LPS and palmitate synergistically stimulate sphingosine kinase 1 and increase sphingosine 1 phosphate in RAW264.7 macrophages. J Leukoc Biol..

[46] Kitatani K, Idkowiak-Baldys J, Hannun YA (2008). The sphingolipid salvage pathway in ceramide metabolism and signaling. Cell Signal.

[47] Schilling JD, Machkovech HM, He L, Sidhu R, Fujiwara H, Weber K (2013). Palmitate and lipopolysaccharide trigger synergistic ceramide production in primary macrophages. J Biol Chem.

[48] Salazar-Onfray F, Lopez MN, Mendoza-Naranjo A (2007). Paradoxical effects of cytokines in tumor immune surveillance and tumor immune escape. Cytokine Growth Factor Rev.

[49] Bolpetti A, Silva JS, Villa LL, Lepique AP (2010). Interleukin-10 production by tumor infiltrating macrophages plays a role in human papillomavirus 16 tumor growth. BMC Immunol.

[50] Chiba N, Masuda A, Yoshikai Y, Matsuguchi T (2007). Ceramide inhibits LPS-induced production of IL-5, IL-10, and IL-13 from mast cells. J Cell Physiol.

[51] Corvinus FM, Orth C, Moriggl R, Tsareva SA, Wagner S, Pfitzner EB (2005). Persistent STAT3 activation in colon cancer is associated with enhanced cell proliferation and tumor growth. Neoplasia.

[52] Banerjee K, Resat H (2016). Constitutive activation of STAT3 in breast cancer cells: a review. Int J Cancer.

[53] Sato N, Tsuruma R, Imoto S, Sekine Y, Muromoto R, Sugiyama K (2005). Nuclear retention of STAT3 through the coiled-coil domain regulates its activity. Biochem Biophys Res Commun.

[54] Kamran MZ, Patil P, Gude RP (2013). Role of STAT3 in cancer metastasis and translational advances. Biomed Res Int.

[55] Murray PJ (2005). The primary mechanism of the IL-10-regulated antiinflammatory response is to selectively inhibit transcription. Proc Natl Acad Sci USA.

[56] Yeh JE, Toniolo PA, Frank DA (2013). Targeting transcription factors: promising new strategies for cancer therapy. Curr Opin Oncol.

